# Commentary: Caring for a violent relative with severe mental illness: a qualitative study

**DOI:** 10.1177/1744987120938347

**Published:** 2020-09-07

**Authors:** Alan Simpson

**Affiliations:** Professor of Mental Health Nursing, Florence Nightingale Faculty of Nursing, Midwifery and Palliative Care, and Health Services and Population Research, King’s College London, London, United Kingdom

Many years ago, I was commissioned by a mental health charity in the United Kingdom to undertake a research study with family carers of people with severe mental illness. I arranged interviews and focus groups with carers in different parts of the country and travelled around meeting people and hearing their stories. It was both heart breaking and illuminating.

Repeatedly, I heard how families had struggled to support their relatives and many of the challenges they faced. People told me about the love and devotion they experienced in relation to their relative and the difficulties experienced. I heard of ingenious approaches people had devised to manage and cope with the testing situations they encountered and of the times they were left drained and defeated.

People’s stories echoed what I had read in the literature on the enormous emotional burden that carers experienced but also the less-often reported, almost spiritual impact of facing and dealing with the most testing of times. Over time, family carers frequently faced mental and physical exhaustion, loss of employment and finances, an end to holidays away and tensions and disappointments with friends and family who failed to understand or appreciate what they were dealing with. Frequently, carers’ own wellbeing and physical health deteriorated and anxieties around the future needs and safety of their relative dominated their thinking.

What often made these experiences even more challenging was the frustration and disappointment of dealing with mental health services; services they assumed would be there to help them and their relative. More often, they found doors closed to them when they asked for advice or support. Their first-hand experiences and concerns were ignored or dismissed by healthcare staff. Accessing services was problematic and far too often impossible. On more than one occasion I came away from interviews deeply upset and disappointed at what I had heard.

Some years later when my own father developed dementia, I saw my mother experiencing similar exhausting demands and frustrations. Just like many of the carers I had interviewed before, after the initial resistance to identifying with the label ‘carer’ (‘I am his wife, I love him; it’s what you do’), my mother found the best support and helpful information from other carers in weekly carer support groups.

The stories I heard stimulated me to ensure that understanding the experiences and frustrations of carers, and how best to support families, was included in the education of mental health nursing students. I worked alongside carers to develop interactive workshops, where we drew on their experiences and those of people I had interviewed. We also highlighted the excellent Triangle of Care, developed to promote the involvement of carers in mental health service design and delivery (Worthington et al., 2013).

There is a paucity of research on the experiences of carers and obtaining funds to undertake large-scale studies is difficult. Our own studies of care planning found carers still often on the side-lines when care plans are developed (Simpson et al., 2016) and rarely included in risk assessment and management (Coffey et al., 2017). This paper on the experiences of carers caring for mentally ill family members who are sometimes violent echoes many previously reported concerns while identifying new areas for consideration and attention. It is well worth a read.
